# Crustal movements due to Iceland’s shrinking ice caps mimic magma inflow signal at Katla volcano

**DOI:** 10.1038/srep10285

**Published:** 2015-05-20

**Authors:** Karsten Spaans, Sigrún Hreinsdóttir, Andrew Hooper, Benedikt Gunnar Ófeigsson

**Affiliations:** 1 COMET, School of Earth and Environment, University of Leeds, Leeds, United Kingdom; 2 Nordic Volcanological Center, Institute of Earth Science, University of Iceland, Reykjavík, Iceland; 3 Icelandic Meteological Office, Reykjavík, Iceland

## Abstract

Many volcanic systems around the world are located beneath, or in close proximity to, ice caps. Mass change of these ice caps causes surface movements, which are typically neglected when interpreting surface deformation measurements around these volcanoes. These movements can however be significant, and may closely resemble movements due to magma accumulation. Here we show such an example, from Katla volcano, Iceland. Horizontal movements observed by GPS on the flank of Katla have led to the inference of significant inflow of magma into a chamber beneath the caldera, starting in 2000, and continuing over several years. We use satellite radar interferometry and GPS data to show that between 2001 and 2010, the horizontal movements seen on the flank can be explained by the response to the long term shrinking of ice caps, and that erratic movements seen at stations within the caldera are also not likely to signify magma inflow. It is important that interpretations of geodetic measurements at volcanoes in glaciated areas consider the effect of ice mass change, and previous studies should be carefully reevaluated.

Katla volcano in Iceland has had several periods of increased activity^1^ after its large eruption in 1918, the 21st since settlement of Iceland in the ninth century AD^2^. Interaction between magma and the overlying ice cap causes Katla eruptions to be explosive, with jökulhlaups (glacial outburst floods) flowing from beneath the glacier. A vast jökulhlaup from an outlet glacier on the east side of the icecap accompanied the 1918 eruption, with the water reaching heights of up to 25m (ref. 3 ). Since 1918, Katla has shown several periods of increased activity. Three major jökulhlaups took place (in 1955^4^, 1999^5^ and 2011), which were possibly the result of small eruptions that did not break through the ice cap, but may also be linked to geothermal activity.

Abutting Katla to the west is Eyjafjallajökull volcano, which caused disruption of air traffic in north-west Europe during a summit eruption between April and May 2010^6^. Eyjafjallajökull had three documented historic eruptions prior to 2010. Interestingly, all three eruptions were followed by an eruption of Katla within two years^2^,^7^,^8^. The possible connection between eruptions of the two volcanoes, combined with the high seismic activity, the historic eruption frequency of Katla and lack of recent eruptions, has led to Katla being considered as a likely volcano to erupt in the coming years^8^.

Three continuous GPS stations were installed in south Iceland in 1999 and 2000, in response to the 1999 jökulhlaup and episodes of seismic unrest at Katla, as well as two intrusions beneath Eyjafjallajökull in 1994 and 1999^9^,^10^. From 2000, two of these stations (SOHO and HVOL), located on the southern flank of Katla’s central volcano, showed horizontal movement outward from the volcano^11^. A nearby station (THEY), located on Eyjafjallajökull’s southern flank, did not show these horizontal movements. Together with movements observed at two benchmarks on nunataks protruding through the ice cap and seismicity beneath Katla, the horizontal movements at the SOHO and HVOL were interpreted as being due to increased pressure in a magma chamber^12^, located beneath the caldera at a depth of around 1.5 km b.s.l.^13^.

Another possible explanation for the movements of the two flank GPS stations is deformation resulting from ice unloading. More than 10% of Iceland is covered in ice^14^, and the majority of these ice caps have been losing mass since ~1890^15^, causing a widespread uplift signal due to Glacial Isostatic Adjustment (GIA)^16^,^17^,^18^. Besides the uplift signal, GIA also results in a horizontal movement away from the unloading source. The possibility of GIA causing the horizontal motions at SOHO and HVOL has been previously investigated based on modelling of ice mass loss over Katla alone. This study reached the conclusion that ice mass loss at Katla could not generate sufficient horizontal motion to explain the movements seen at the GPS stations^19^.

Here we use a combination of satellite radar interferometry and GPS measurements to investigate the outward movement of the southern flank observed from 2000^8^,^12^. The combined InSAR and GPS dataset provides a much improved spatial sampling density, revealing spatial patterns in the deformation field. These spatial patterns are key to pinpointing the source of any deformation.

## Results

**Figure 1:**
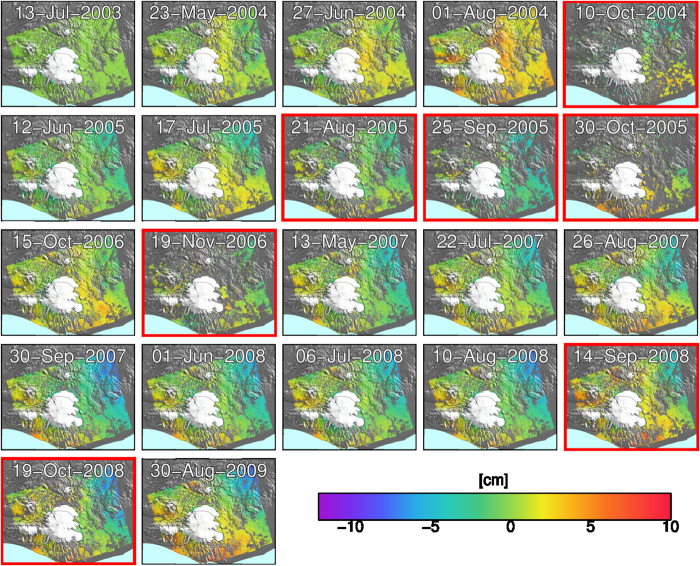
Time series of unwrapped interferograms. Each image shows the cumulative phase change with respect to the first image (July 13, 2003). The images surrounded by a red box are those identified as having lower overall coherence, due to snow or long baselines. These images are processed using the extension to StaMPS described in Hooper *et al*.^21^, and therefore only contain a subset of the points selected in the regular images. Negative phase differences indicate line of sight shortening (i.e. movement towards the satellite). The maps were created using the public domain Generic Mapping Tools software package.

**Figure 2:**
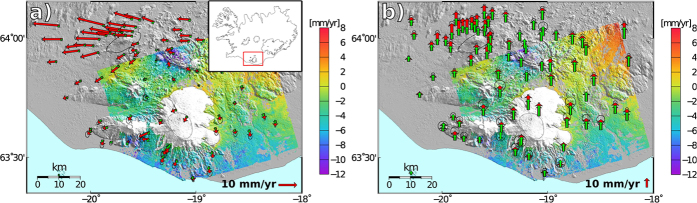
(**a**) Horizontal and (**b**) vertical GPS (red) and GIA model predictions (green) velocity field, plotted on the InSAR velocity. GPS velocities are relative to the ITRF08 Eurasian fixed reference frame. Positive InSAR velocities indicate movement towards the satellite. The error ellipses on the GPS give the 95% confidence region. The inset in panel **a**) shows the outlines of Iceland, and the red box shows the outlines of panels a) and **b**). The map, including the inset, was created using the public domain Generic Mapping Tools software package.

We used a set of 22 ESA Envisat images acquired along track 87, to form 21 interferograms covering the period between July 2003 and August 2009, and estimated an average velocity during this time period using the StaMPS software^20^,^21^. We developed an extension to StaMPS that aims to minimise the detrimental effects that low coherence images have on the number and quality of selected points^21^. A short overview of the StaMPS extension can be found in the Methods section. The resulting timeseries of unwrapped interferograms is shown in Fig. 1. From the timeseries, we generated a velocity map by estimating a constant rate for each selected point (Fig. 2).

Furthermore, we analyzed all available campaign and continuous GPS data from south Iceland between 2001 and 2010^8^,^12^,^16^,^22^,^23^ to estimate a velocity field for the region (Fig. 2, Methods). One of the main features present in the horizontal GPS velocity field is the dominant westward movement in the north-west of the scene (Fig. 2a). This area is located in the South Iceland Seismic Zone, a transform zone between the Reykjanes Peninsula in the west of Iceland, and the Eastern Volcanic Zone commencing north of Katla. The area is moving mostly with the North American tectonic plate^23^, explaining the westward movements with respect to the Eurasian plate.

**Figure 3:**
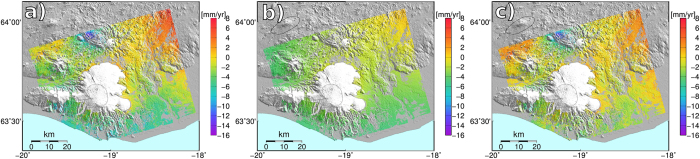
Average InSAR velocities for the period 2003-2009. (**a**) InSAR velocities corrected for the oscillator frequency drift (See Methods). (**b**) GIA model velocities projected on the radar LOS. (**c**) InSAR velocities corrected for local oscillator drift and GIA. The map was created using the public domain Generic Mapping Tools software package.

Both the vertical GPS and the InSAR velocity fields show an uplift signal of increasing magnitude towards the north-east of the scene (Fig. 2), likely due to ice unloading of Vatnajökull glacier. We used the results of a finite element model^17^ to remove the contribution of the GIA to the InSAR and GPS signal (Figs. 2 and 3). The model assumes an ice model, and constrains a vertically variable rheology using vertical GPS velocities between 1993 and 2004. The best earth model has 2 layers, a 35 km thick elastic layer, overlying a visco-elastic layer with viscosity of 10^19^ Pa s^17^.

The GIA model underpredicts the measured velocities, especially in the horizontal components (Figs. 2 and 3). The model was constrained using data in the decade before the deformation measurements used in this study. Mass balance measurements of the ice caps in Iceland have shown that after 1997, the ice caps started to lose ice at an increased rate^15^. This would have lead to an increase in GIA uplift in the years to follow^24^. More importantly, it has been observed in previous studies that the GIA models underpredict the magnitude of the horizontal velocity in general^16^,^18^. Specifically, residual horizontal GPS velocities throughout Iceland between 1993 and 2004 are often more than twice the magnitude of the actual modelled GIA signal^16^, consistently throughout Iceland. We therefore attribute the large residuals in the horizontal velocities around Katla to the systematic underprediction of these FEM models in the horizontal, possibly aggravated by the increased melting in recent years.

**Figure 4:**
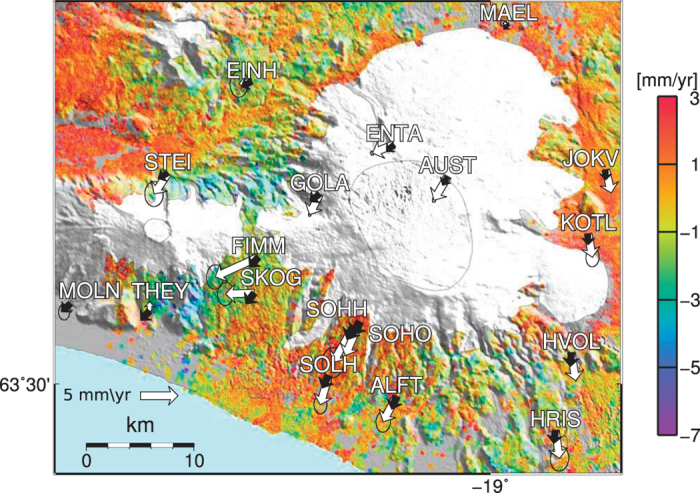
Close-up view of the Mýrdalsjökull area showing the velocity estimates of the InSAR results after the removal of the GIA model, between 2003 and 2009. Positive velocities indicate movement towards the satellite. Overlain on the InSAR velocities are the GPS velocity vectors between 2001 and 2010 in white, and the model velocity vectors in black. GPS error ellipses shows the 95% confidence region. The map was created using the public domain Generic Mapping Tools software package.

After correction for the GIA, we find no signals in the InSAR and GPS velocity fields that would indicate significant magma movements beneath the volcano. A close-up view of Katla (Fig. 4) shows that around the edges of some of the outlet glaciers there are increased movements in the InSAR residual velocities. As these increased movements follow the edge of individual outlet glaciers closely, they are likely due to increased melting of low altitude parts of the outlet glaciers, something not captured in the GIA model^17^. Figure 4 also shows the horizontal GPS and GIA model velocities. The displacement rates at the campaign stations on the ice cap, as well as those at the continuous stations SOHO and HVOL on the south flank, agree in terms of direction with the GIA model. This suggests that the horizontal movements are most likely due to ice mass loss at the Mýrdalsjökull icecap partially covering Katla and the large Vatnajökull icecap to the east.

One of the reasons that the horizontal movements at SOHO and HVOL stations were attributed to pressure increase in the magma chamber of Katla was that a third continuous station, THEY, did not show this south-southwest ward movement. However, the inclusion of additional campaign GPS stations and the InSAR results (Fig. 4) clearly shows that in fact it was THEY, and other stations on the south flank of Eyjafjallajökull, that behaved differently to the regional trend. The horizontal GPS, vertical GPS and the InSAR velocities alike show a region of subsidence and horizontal movement towards a point on the south flank of Eyjafjallajökull. The position of this signal matches that of deformation resulting from two intrusions beneath Eyjafjallajökull in 1994 and 1999^9^,^10^, suggesting cooling of the intruded lava as being the cause of this contraction signal.

**Figure 5:**
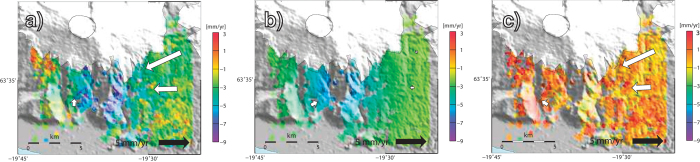
Model of the contracting signal on the south flank of Eyjafjallajökull. Panel **a**) shows the resampled InSAR velocity, overlain by the gps vectors in white, panel **b**) shows the model results projected on the radar line-of-sight, as well the horizontal model predictions in white, and panel **c**) shows the residual velocity after removing the model from the InSAR velocity, overlain by the residual gps vectors. The maps were created using the public domain Generic Mapping Tools software package.

To evaluate if the signal can be explained by a contraction of a sill, we have modelled it as a penny shaped crack^25^. We fixed the radius and the position of the sill to closely resemble modelling results for the intrusion in 1999^10^, and varied the excess pressure (see Methods). The best fitting model is shown in Fig. 5. The InSAR velocities are fit remarkably well by the model, indicating that the source of the contraction is the same as, or closely related to, the 1999 intrusion. The fit to the horizontal GPS vectors is not as good. The GPS vectors are however far more sensitive to the residual GIA signal discussed above, and presumably contaminated by it.

## Discussion

Pinel *et al.*^19^ applied an analytical model of long term ice unloading to evaluate the effect of thinning of the Mýrdalsjökull icecap and concluded that it could not explain the observed horizontal movements at the GPS stations around Katla volcano. The influence of the ice mass loss of the larger Vatnajökull icecap was however neglected, while more recent visco-elastic finite element models including all ice caps show that there is in fact a significant influence from Vatnajökull in south Iceland (Fig. 2)^16^,^17^.

**Figure 6:**
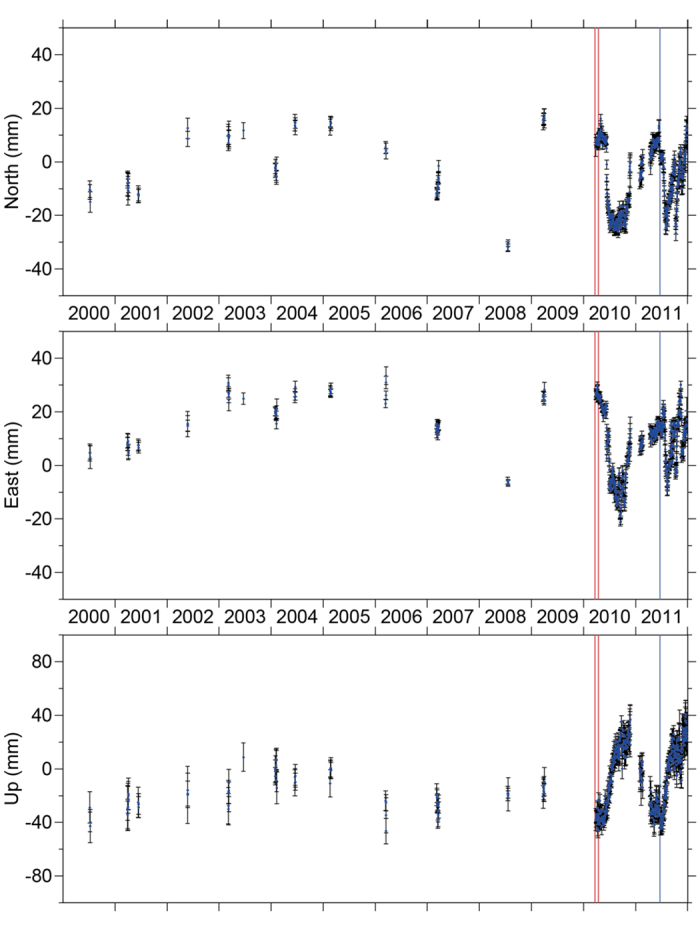
GPS time series for the Austmannsbunga station, located on the edge of the Katla caldera. The red vertical lines indicate the onset of the two eruptive events of Eyjafjallajökull in 2010, and the blue vertical line indicates the jökulhlaup at Katla in 2011. Linear trends have been removed from the time series.

Observations of erratic behaviour at GPS sites on the caldera rim cast doubt on whether there was significant deformation resulting from pressure increase in the Katla magma chamber in the period 2000-2004. Since they became continuous in 2010, large annual variations have been observed (Fig. 6). Superimposed on the long term ice unloading signal, both periodic signals, due to primarily seasonal snow loading, and individual excursions can be seen. These large variations in displacement throughout the year are on the same order of magnitude as the inferred velocity changes, which means that it is difficult to identify the cause of the displacements observed in the campaign GPS measurements between 2000 and 2004, which were in the order of 2–3 cm^12^. The high frequency with which the current displacements at AUST vary means that estimated deformation rates were highly affected by the timing of the campaign measurements. Therefore, the earlier hypothesis of increased pressure in the magma chamber likely represents an over-interpretation of limited measurements in both time and space, and other processes like snow and ice unloading or water pressure variations at the base of the icecap are at least as likely to be the cause of the observed movements around Katla.

It is important to note that our results do not rule out the possibility of magma accumulation beneath Katla during the period 2001–2009. We have shown that horizontal motions at stations outside of the icecap, previously attributed to magma accumulation, are more likely to be caused by ice unloading. If there was any pressure increase in the magma chamber, significant deformation did not reach outside of the icecap, thus representing far less volume than previously inferred^12^. It is possible that small deformations could have been obscured by the GIA signal and short term loading effects.

The contraction signal present in the InSAR velocities on the south flank of Eyjafjallajökull are fit well by a contracting penny shaped sill. The systematic horizontal discrepancy between observations and GIA models is still present in the residual horizontal GPS vectors. This explains in part the mismatch between the horizontal GPS and the model. It demonstrates the importance of evaluating the effects of long-term ice unloading when interpreting volcano deformation around ice covered volcanoes. However, the direction of the residuals do deviate from the direction predicted by the GIA model (Fig. 4). This could possibly be explained by a difference in geometry from the penny shaped crack model used, caused perhaps by rapid initial cooling of the edges leading to prolonged cooling of a more spheroidally shaped magma body. We cannot, however, rule out the possibility of other processes affecting the GPS observations on Eyjafjallajökull’s southern flank.

Our results show that there is no significant deformation related to inflow of magma between 2001 and 2010, and therefore Katla might not be as primed to erupt as previously thought. However, as the current century long repose period is almost twice the average repose time of the volcano, and seismic activity has been high^1^,^12^, the threat of a Katla eruption cannot be disregarded. Our results show that horizontal deformation due to GIA in Iceland is much larger than previously thought, and that this can lead to erroneous interpretations of data. It is vital to take the effect of GIA into account when interpreting future GPS data in Iceland and other glaciated areas around the world.

## Methods

To form the interferograms, we used scripts from the ROI_PAC^26^ and DORIS^27^ software packages. Topographic phase was removed using a 25 m posted digital elevation model from the Icelandic Geo-Survey. We used the StaMPS method to do the timeseries analysis^20^, with the extension described in 21 to minimize the loss of PS points due to low coherence images, e.g. due to snow cover.

The extension to StaMPS selects a sufficient number of interferograms of high coherence (usually summer acquisitions with low to average perpendicular baselines) to perform a reliable StaMPS PS analysis. Performing the standard StaMPS PS analysis on these high coherence interferograms results in a set of PS points. For the remaining, low coherence interferograms, each PS point is then analysed on an interferogram by interferogram basis, retaining the PS point in that interferogram only if it remains sufficiently coherent. This results in a subset of the original PS points for each low coherence interferogram. Although this yields a different set of PS points for each low coherence interferogram, all PS points are present in the higher coherence interferograms. This allows the 3D unwrapping algorithm used in StaMPS to be used with only minor adaptation.

It has long been known that Envisat images suffer from a systematic ramp^28^, which has recently been shown to be caused by a drift in the local oscillator frequency of the satellite^29^. The linear ramp caused by this drift is removed using the following approximation, derived from an empirical study^29^:


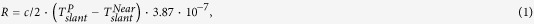


where *R* is the apparent ramp in m yr^−1^, *c* is the speed of light, *T*_*slant*_ indicates the two way slant travel time, the superscript *P* indicates the current pixel and the superscript 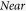 indicates the near-range pixel.

For the GPS processing, we analysed all available campaign and continuous GPS data from south Iceland between 2001 and 2010 to estimate a velocity field for the region. We excluded all data from May 2009 within 10 km of Skógaheidi on the southeast flank of Eyjafjallajökull, due to intrusive activity leading up to the 2010 Eyjafjallajökull eruptions^6^. The GPS data were analyzed using the GAMIT/GLOBK version 10.4 (see T.A. Herring, R.W. King, and S.C. McClusky. *GAMIT reference manual*, **v10.4**, Massachusetts Institute of Technology, October 2010), using available International GNSS Service (IGS) 2008 absolute elevation and azimuth dependent phase center corrections for receiver antennas and ocean-loading model FES2004. We used IGS orbit and Earth orientation parameters as a-priori constraints and estimated adjustments to them during the analysis as well as estimating daily coordinates for the GPS sites. We analyzed the data with a set of 150 selected global reference stations and used GLOBK to estimate the velocity field in a fixed ITRF08-Eurasia reference frame^30^.

We modelled the contraction signal south of Eyjafjallajökull as a penny shaped crack^25^. We fixed the center of the crack to –19.58 longitude and 63.58 latitude, at a depth of 5.7 km and a radius of 2.5 km. We varied the excess pressure drop between 1 · 10^7^ and 5 · 10^8^ Pa. We evaluated the best fitting model compared to the residual InSAR velocities based on the residual sum of squares, weighted by the inverse of the covariance matrix. The best fit model had an excess pressure drop of 3.2 · 10^8^ Pa.

## Additional Information

**How to cite this article**: Spaans, K. *et al.* Crustal movements due to Iceland's shrinking ice caps mimic magma inflow signal at Katla volcano. *Sci. Rep.* **5**, 10285; doi: 10.1038/srep10285 (2015).
