# Corrigendum: Crustal movements due to Iceland’s shrinking ice caps mimic magma inflow signal at Katla volcano

**DOI:** 10.1038/srep33010

**Published:** 2016-09-14

**Authors:** Karsten Spaans, Sigrún Hreinsdóttir, Andrew Hooper, Benedikt Gunnar Ófeigsson

Scientific Reports 5: Article number: 1028510.1038/srep10285; published online: 05 20 2015; updated: 09 14 2016

This Article contains typographical errors in the Methods section,

“We varied the excess pressure drop between 1 · 10^7^ and 5 · 10^8^ Pa. We evaluated the best fitting model compared to the residual InSAR velocities based on the residual sum of squares, weighted by the inverse of the covariance matrix. The best fit model had an excess pressure drop of 3.2 · 10^8^ Pa”.

should read:

“We varied the excess pressure drop between 1 · 10^4^ and 5 · 10^5^ Pa. We evaluated the best fitting model compared to the residual InSAR velocities based on the residual sum of squares, weighted by the inverse of the covariance matrix. The best fit model had an excess pressure drop of 3.2 · 10^5^ Pa”.

